# 

**Published:** 1921-06

**Authors:** 


MARCH AND JUNE, 1921.

*Vol.* XXXVIII. *No.* 142.

### THE BRISTOL MEDICO-CHIRURGICAL JOURNAL

Library of Congress
AUG 5 1921
537435

PUBLISHED UNDER THE AUSPICES OF

*THE BRISTOL MEDICO-CHIRURGICAL SOCIETY.*

*Editor:* P. WATSON-WILLIAMS, M.D.,
WITH WHOM ARE ASSOCIATED
JAMES SWAIN, C.B., C.B.E., M.S., M.D.,
J. LACY FIRTH, M.S., M.D., CAREY COOMBS, M.D.
J. A. NIXON, C.M.G., M.D., *Assistant Editor.*
*Editorial Secretary*: A. L. FLEMMING, M.B., Ch.B.

BRISTOL: J. W. ARROWSMITH LTD.
LONDON: SIMPKIN, MARSHALL, HAMILTON, KENT & Co. LTD.

*Price Three Shillings net.*

